# Supplementary material for: Are carbon emissions trading and green financial instruments synergistic? -Comprehensive quantitative research based on content analysis
Source: PLoS One. 2024 Mar 7;19(3):e0298601. doi: 10.1371/journal.pone.0298601 (PMC10919601; doi:10.1371/journal.pone.0298601)
Supplement: S1 Data — (ZIP) [file pone.0298601.s001.zip › Data/地级市Z_F工作报告.zip!/║╙▒▒/║Γ╦«╩╨/└┤╫╘╣1⁄2╓┌║┼í╛┬φ┐╦╩2╛▌═°í┐_2007.docx]

各位代表：

现在，我代表县人民政府，向大会报告本届政府四年的工作，对下届政府和今年的工作提出建议，请予审议，并请县政协各位委员和列席会议的同志提出意见。

一、过去四年工作回顾

过去的四年，是枣强发展史上的关键时期。四年来，在中共枣强县委的正确领导下，在县人大、县政协的监督支持下，我们同全县广大干部群众一道，坚持以邓小平理论和“三个代表”重要思想为指导，牢固树立和落实科学发展观，攻坚克难，与时俱进，经济社会各项事业全面发展，圆满完成了既定的各项目标任务。

综合实力明显增强。全县生产总值由2002年的36.8亿元增加到2006年的42.9亿元；财政收入由2002年的1.2亿元增加到2006年的3.6亿元，年均增长48.2%；全社会固定资产投资累计达到89.7亿元，其中工业投入60.2亿元。先后被市委、市政府评为“工业发展先进县”、“工业基地建设先进县”、“重点项目建设先进县”、“财政增收先进县”和“对外开放先进县”。

工业发展步伐加快。2006年，实现工业总产值55.3亿元，规模以上工业实现利税14亿元，按可比口径，年均分别增长9.5 %和11.2%。皮毛、玻璃钢两大产业的发展增速年均超过20%，以输送机械、燃气调压器为主的机械制造业发展增速年均达到50%，已经成为工业发展的第二梯队。三大产业上交税金2.9亿元，占全部财政收入的80％。科技进步成效显著。共开发新产品39项，创市级以上名牌产品6项，其中恒润牌玻璃钢管道被评为中国名牌产品，“弘飞”、“冀峰”2项商标被评为省著名商标。项目建设取得新进展。共有46个项目挤入省市计划笼子，争取建设用地指标2385亩，位居全市前列。先后开工建设固定资产投资300万元以上的项目207个，完成投资45.7亿元。发展平台不断拓展。玻璃钢工业园区投资900万元，完成了7条路街建设和绿化、美化、亮化工程，建成区面积由1平方公里发展到现在的近4平方公里，被国家发改委和省政府批准保留为省级工业园区。2006年，区内企业实现总产值18.3亿元，利税2.8亿元。大营皮毛工业区以及新屯、秀屯、马屯工业小区建设全面推进，以县城、大营两大园区为龙头、产业结构合理搭配、准入门槛高低有别、投资主体互为补充的园区发展新格局正在形成。

改革开放取得突破。全县国有集体企业改制面达到80%，盘活资产2.45亿元。深入开展了“招商项目年”活动，不断强化招商责任，创新招商方式，优化招商环境，取得了新的成效。全县新注册三资企业9家，累计利用外资2664万美元、引进内资14.8亿元、技术331项、人才4505名，引进县外企业198家。曼吉克工艺玻璃、无污染清洁制浆、华联超市等一批大项目相继落户我县。大营国际皮草交易中心成为我县目前引进的最大单体项目。连续成功举办了中国？大营国际皮草交易会和裘皮制品订货会，枣强的知名度得到新的提升。

“三农”工作力度加大。认真落实农业税减免、粮食直补、良种补贴、农机具购置补贴等惠农政策，累计发放各项补贴6000万元，减免农业税4172万元。农业基础设施建设扎实有效。累计新打更新机井2300多眼，铺设防渗管道40万米，新增有效灌溉面积20多万亩。2006年，全县粮食产量达到31万吨，有林面积达到21.3万亩，以奶牛、水貂、狐狸、獭兔为主的特色养殖出栏达到40万头，分别比2002年增长23% 、103%和370%。文明生态村建设稳步推进。全县已有116个文明生态村通过验收，涌现出东岳庄、八里庄、肖张村等一批省市创建典型。

城乡面貌正在改观。城市规划体系基本形成，“一渠两岸和四条主干街道”的控制性详规已经完成，描绘了城市长远发展的宏伟蓝图。城市建设迈出新步伐，投资1600万元，对建设路、裕华路、东环北路进行了拓宽改造，硬化了17条小街小巷。投资3100万元，对县城污水管网和卫千渠县城段进行了改排改造。果蔬中心市场投入运营，森林公园框架基本形成，城市花园建设进展顺利，首期12栋住宅楼交付使用。第二水厂有望今年国庆节前联网运行。县城污水处理厂前期准备工作正在加紧进行。城市管理得到加强，绿化、亮化、美化、净化水平逐步提高。重视和支持小城镇建设，大营镇先后投资830万元，完成了人民街、经贸路、西环路和火车站广场等重点工程建设。大营污水处理厂正在进行设备安装调试。县城和大营镇先后被评为省级环境优美小城镇。强力推进城乡公路建设，投资1.5亿元，完成了邢德路加宽改建、武馆线大修、肃临线加宽改建、张唐路、枣景路一期等一批重点工程，全县实现通车里程达到750公里，比2002年净增400多公里。投资6314万元，完成了252个村350.8公里村村通工程，惠及群众20万人。投资6300万元，对县城电网进行了改造，新建110千伏变电站1座、35千伏变电站3座，输变电总容量达到24万千伏安。

人民生活继续改善。城镇居民人均可支配收入由5698元增加到7900元，年递增9.7%；农民人均纯收入由2939元增加到3757元，年递增7%。城乡居民储蓄余额达到44.3亿元，比2002年增长69%。社会保障体系逐步完善，养老、医疗、失业、工伤、生育五大险种平稳运行，覆盖面不断扩大。成为全市唯一一家由省级直接调剂养老保险金试点县，在全市率先尝试国有破产企业养老保险欠费核销工作和开辟公益性就业岗位。累计争取上级调剂金4618万元，保证了2670名离退休人员养老金按时足额发放。健全完善了城镇低保，启动了农村低保，累计发放救助资金800万元。较好地解决了乡镇教师工资拖欠等历史遗留问题。加强劳务输出，每年向京津石等地输出劳力8000余人次。为民承诺办理的33件实事如期完成，解决了一批与人民群众生产生活密切相关的实际困难和问题。

社会事业全面进步。科技工作成绩突出。校县合作和科技成果引进推广成效显著。先后建成了国家级“复合材料生产力促进中心”和省级玻璃钢、裘皮、燃气调压器研发中心，被授予“全国科技工作先进县”和“国家科技富民强县试点县”。教育事业展露新姿。投资8000多万元，实施中小学危房改造工程，新建翻建校舍3.4万平方米，维修3.5万平方米，基本消除了中小学危房。投资3000余万元，完成了枣中新校区建设，办学条件明显改善，教育质量逐步提高，高考成绩连年提升。卫生工作先开新河。在全省率先推行了新型农村合作医疗，努力提高农民参合率、受益率和救治率。四年来，共救助农民52万人次，发放补助资金2500万元。精神文明与民主法制建设进一步加强。培育选树了全国优秀共产党员林秀贞等一批典型，群众性精神文明创建活动进一步深化。和谐枣强建设扎实推进。深入开展社会治安综合治理，有效化解了各种不安定因素，扎实开展严打整治斗争，有力打击了各种违法犯罪活动，全县政治安定、社会稳定、人民群众安居乐业的大好局面得到巩固和发展。计划生育、国土、统计、审计、物价等工作圆满完成任务，国防动员、广播电视、体育、档案等社会各项事业取得新进展。

自身建设不断加强。坚持科学决策、民主决策、依法决策，积极推行政务公开制度、事前公示制度和听证制度，对重大问题，认真听取专家学者和群众的意见，尽可能减少决策的失误和偏差。坚持谋全局、抓大事、重实干、求实效，努力转变职能，改进作风。坚持主动接受人大的法律监督、工作监督和政协的民主监督，人大代表建议、政协委员提案办结率达到100%。坚持把优化发展环境放在突出位置，深入开展了行政权力公开透明运行工作，对重点项目和重点企业实行了“无干扰”挂牌保护，营造了亲商、安商、富商的投资环境和创业环境。

总之，过去的四年，是工业经济提速发展，综合实力明显增强的四年；是农村经济稳步推进，农民收入持续增长的四年；是基础设施逐步完善，城镇面貌明显变化的四年；是社会事业全面进步，群众得到实惠明显增多的四年。四年历程很不平凡，成绩来之不易。所有这一切，无不凝聚着全县干部群众奋发图强的不懈努力，无不凝聚着历届班子破难奋进的艰辛汗水，无不凝聚着社会各界一以贯之的鼎力支持。在此，我代表县政府，向全县人民，向给予政府工作支持和监督的人大代表、政协委员和离退休老同志，向所有关心、支持枣强发展的各界人士和朋友们，表示崇高的敬意和衷心的感谢！

在看到成绩的同时，我们也要清醒地看到，我县经济和社会发展的形势依然严竣，影响枣强跨越发展的因素还很多：一是经济实力不强，发展质量不高，工业化和城镇化进程滞后，财政收支矛盾突出，作为欠发达县份的基本特征尚未根本改变；二是工业发展结构性矛盾突出，主导产业层次较低，综合竞争力不高，产品档次亟待升级，对部分皮毛企业违法违规行为进行治理整顿的任务还非常繁重，土地、环保等制约发展的因素尚未根本消除；三是南北差距、乡镇差距较大，社会事业发展欠账较多，发展不平衡的状况尚未根本扭转；四是关系民生的矛盾和难题日益显现，安全生产还存有隐患，影响社会和谐稳定的压力尚未根本缓解；五是发展环境不尽人意，一些政府部门和工作人员缺乏大局意识、服务意识、配合意识和责任意识，思想不够艰苦，作风不够深入，工作缺乏主动性、积极性和创造性。我们必须高度重视这些问题，以科学务实的态度，采取更加有力的措施，认真加以解决。

在实践中我们深刻体会到：谋好枣强的局、干好枣强的事，必须坚持以科学发展观为统领，全力抓好发展第一要务，积极探索具有时代特征、遵循客观规律、符合枣强实际的发展路径，这是我们的执政之责、富民之道，强县之策；必须坚持发展为了人民，发展依靠人民，发展成果由人民共享，努力解决好人民群众最关心最现实最直接的利益问题，充分调动全县人民的积极性创造性；必须坚持深化改革、扩大开放，把激发内力与借助外力结合起来，不断拓展发展新空间、创造发展新优势；必须坚持正确处理改革发展稳定的关系，在社会稳定中推进改革发展，在改革发展中促进和谐稳定。

二、今后五年发展设想

各位代表，今后五年，是我县贯彻落实科学发展观、构建社会主义和谐社会的重要时期。纵观面临形势，把握历史方位，我们已站在一个新的更高起点，置身于一个充满机遇和挑战的新阶段。

当前，我县面临着难得的发展机遇。国际、国内发展环境趋好，产业结构大调整和产业大转移步伐加快，区域合作日益紧密，河北建设沿海经济社会发展强省重大战略的实施，为我县跨越发展提供了良好的宏观环境。改革开放、科技创新发展动能汇聚，南资北移趋势日益明显，为我县跨越发展创造了有利条件。我县自身的条件也正在发生着非常有利的变化，全县上下团结一心，务实求进，“谋发展、促跨越、重实干”已成为各级干部的自觉行动，各项事业蒸蒸日上，蓬勃发展，更为我县跨越发展奠定了坚实基础。同时，我们也面临巨大的挑战。既要提高发展质量，又要加快发展速度，推进科学发展的任务更加艰巨；既要应对国际竞争国内化的新趋势，又要应对国内竞争区域化的新情况，面临的竞争更为激烈；既要聚精会神抓发展，又要切切实实促和谐，对执政能力提出更高要求。

站在新的起点，谋划枣强发展，必须进一步解放思想，开拓奋进，始终保持振奋的精神和良好的作风。特别是面对新的形势和任务，一定要有敢于率先、攻坚破难的勇气，冲破思想桎梏，摒弃陈规陋习，在破解难题中加快发展，在化解矛盾中促进和谐。一定要有争创一流、创造卓越的追求，高起点谋划思路，高标准开展工作，高水平推进发展。一定要有昂扬向上、奋发进取的精神，保持干事业的饱满热情，弘扬干实事的务实作风，永不自满，永不懈怠，不断开创各项事业发展新局面。

今后五年政府工作的指导思想和总体要求是：坚持以邓小平理论和“三个代表”重要思想为指导，全面落实科学发展观，围绕“创新实干、跨越发展”这个主题，大力实施工业强县、开放兴县两大战略，着力推进经济结构调整和增长方式转变，着力推进发展平台建设和重大项目的战略支撑，着力推进城镇建设和社会主义新农村建设，着力推进社会事业和民生问题的解决，坚持好中求快，快中见好，实现经济社会的跨越发展。

今后五年我县经济社会发展总的目标是：力争一年有新进展、三年有新突破、五年上新台阶。“一年有新进展”，就是在优化结构、提高效益和节能降耗、保护环境的基础上，全县经济增长的质量和速度有新的提升，2008年财政收入达到历史最好水平。“三年有新突破”，就是人均生产总值超过2000美元，财政收入突破4亿元，提前完成“十一五”主要经济目标。“五年上新台阶”，就是实现财政收入比2007年翻一番以上，工业化、城镇化、市场化水平明显提升，社会事业明显进步，社会主义新农村建设取得明显进展，城乡居民生活质量明显提高。实现上述目标任务，要把握以下几点：

？？更加注重效益优先、好中求快，坚定不移地走科学发展之路。正确处理当前与长远、经济与社会、富民与强县的关系，以工业经济和项目建设为总抓手，注重结构调整，切实转变增长方式，推动经济社会全面协调可持续发展。

？？更加注重富民优先、民生为本，坚定不移地走和谐发展之路。树立以人为本的理念，把富裕人民作为发展的首要任务，在发展中富民，在富民中促进社会和谐。维护和发展根本利益，更加注重切身利益，着力办好群众急需、受益的事情。坚持效率优先，更加注重公平，促进共同富裕。关注生活质量，更加注重满足精神文化需求，促进人的全面发展。

？？更加注重科教优先、求新求进，坚定不移地走创新发展之路。树立创新引领未来的理念，鼓励创新，支持创业，尊重创造。坚持实施科教兴县和人才强县战略，提高自主创新能力，加快建设创新型枣强。坚持改革开放，破除制约发展的体制机制障碍。创新思想理念，创新思维方式，创新工作方法，向创新要发展的动力、发展的资源、发展的空间。

？？更加注重优势优先、重点突破，坚定不移地走跨越发展之路。树立非均衡推进的理念，抢占发展先机，发挥后发优势，力争用相对较短的时间缩小差距，甚至在一些领域实现赶超，实现新的跨越。遵循经济社会发展规律，勇于打破常规，集中力量，突出重点，在关键领域实现突破。着力营造跨越氛围，凝聚跨越合力，以非常之策行跨越之举，以非常之为强跨越之势，以非常之力求跨越之效。

三、关于2007年工作

2007年是新一届政府的开局之年，也是创新实干，跨越发展的转折之年。做好今年的工作，意义十分重大。今年经济发展的主要预期目标是：全县生产总值增长10%以上，全社会固定资产投资增长24%以上，农民人均纯收入增长6%，财政收入完成2.2亿元以上，力争完成得更好一些。

实现以上目标任务，必须全力做好以下六个方面的工作：

第一、着力做强工业经济，强化跨越发展的支撑

工业是财政收入的主要来源，是县域经济的支撑和命脉。坚持走新型工业化道路，大力实施工业强县战略，加快建设具有国际国内重要影响的特色产业发展基地，提高工业经济核心竞争力。

做强做大主导产业。按照“着眼竞争，发挥优势；突出重点，培育体系；适时调整，加快升级；注重环境，提高效益”的总体思路，抓紧研究制定皮毛、玻璃钢两大主导产业的中长期发展规划，进一步完善落实推进两大主导产业发展的配套措施，积极引领两大产业朝着特色化、规模化、集群化的方向阔步前进。绝不能因为自身工作，使辛辛苦苦培育起来的产业在竞争中退化、萎缩，甚至被市场淘汰。要全力做好皮毛业的治理整顿工作，力促税案快查快结。继续毫不动摇地鼓励支持皮毛业发展，充分发挥主观能动性，积极探索新办法，尽快破解制约皮毛业发展的各种症结，促其实现恢复性增长和营皮北扩战略的顺利实施，坚决防止穿新鞋走老路、走弯路。整合现有资源，引导企业树立全新的经营模式和管理理念，进一步改进工艺，增加品种，提高档次，增强市场占有率和竞争力。要加快玻璃钢业结构调整和升级步伐。巩固扩大玻璃钢管道生产优势，建设国内最大的玻璃钢管道基地。同时，加快玻璃钢门窗、洁净板、防风墙、沼气池、风力发电设备等新产品的开发推广，引导企业向专业化、标准化、现代化迈进。围绕发展循环经济，在皮毛硝染、玻璃钢下脚料综合利用等关键技术和瓶颈技术上强力攻关，全力打造绿色、生态产业。以输送机械、燃气调压器为主的机械制造业，提高装备水平，拉长产业链条，尽快壮大规模，提升影响力，为经济发展提供有力支撑。以无污染清洁制浆项目为重点，全力抓好其他高新技术产业的开发和培育。同时，坚持以产权改革为核心，因企制宜，分类实施，加快企业改革改制，复兴、盘活线缆、棉纺、化工等产业，促进传统产业重新焕发生机，逐步构建两业为主、多点支撑、梯次发展的工业经济发展新格局。

做强做大工业园区。园区是产业集群发展的载体和平台。要按照“扩容、提质、增效、共享”的要求，在集中布局、集约布局、优化布局上下功夫，在扩大开放、招商引资、争上项目上做文章，在创优环境、提供服务、强化管理上用实劲，积极引导企业向园区集中，向瓶颈约束最小、综合优势最大的地方转移，向交通便利、环境容量大、用地能满足供应、经营成本低的区域发展。今年，玻璃钢工业区实施“东扩”战略，按照规划，加快水、电、路及绿化、亮化等基础设施建设，完成区域环评，增强集聚功能。力争新建、续建项目10个，完成投资2.9亿元。大营皮毛工业区重点抓好园区现有土地的整合、盘活，确保重点项目建设用地。坚持布局集中、用地集约和产业集聚的原则，进一步完善园区项目建设用地管理办法，严把产业关、环保关、投资强度和税收回报关，坚决杜绝低水平重复建设。加快枣强镇中小企业创业基地、秀屯、新屯、马屯等乡镇工业小区建设，逐步培育成特色鲜明、有影响力和带动力的工业区。

做强做大骨干企业。按照巩固发展、承接引进、整合扩张的思路，筛选30家骨干企业，重点培育扶持，鼓励规模企业实施战略重组，积极推动优势资源向重点企业集中，发展壮大一批主业突出、品牌知名、核心竞争力强的企业集团。力争年产值超亿元企业达到25家，让“铺天盖地”的产业与“顶天立地”的企业相得益彰，相互促进。加强企业家队伍建设，努力培养一支思想解放、观念超前，有知识、有魄力、高素质的企业经营、管理队伍。大力开展创名牌、创名企、创名家活动。以现有国家省级名牌产品、著名商标为导向，加强产品包装和宣传推介，注重品牌经营、保护和宣传。年内力争培育市级以上名品、名标10个。

第二、倾力抓好项目建设，增强跨越发展的后劲

项目建设是经济增长的“发动机”和“推动器”，牵动着经济社会发展全局，而且决定着发展的速度、质量和后劲。县委、县政府继续把今年确定为“招商项目年”，就是要进一步把领导力量向项目倾斜，把工作精力向项目集中，把工作重点向项目转移，举全县之力推进项目建设。重点抓好“三个一批”：一是投产见效一批。对东方恒强、中韩合成新材、巨鑫、远东等在建项目，强化协调调度，加快建设进度，促其早竣工、早见效，尽快形成经济增长点。确保东方恒强清洁制浆一期5万吨6月底竣工投产，力争年内生产能力达到30万吨。二是启动建设一批。对驹王挂车、正信冷轧钢、益海粮油、中科能源风光互补路灯、静冈刮刀等已经具备开工条件的项目，积极做好前期准备工作，落实资金、土地，促其尽早开工建设。确保年内完成投资2.5亿元以上。三是谋划储备一批。着眼长远发展，积极构建与河北太和集团、辽宁红运集团、河北益海粮油公司等大公司、大集团的战略合作关系，推进合作向纵深拓展。聘请权威专家和业内人士，瞄准国家产业政策新导向，立足枣强实际，围绕“大产业、大基地、大园区”，抓紧谋划一批符合国家产业政策、符合新型工业化要求的重大项目。全年力争谋划投资超500万元项目130个，13个挤入省、市重点笼子。

为确保项目建设取得扎实成效，必须全力抓好以下环节。一是破解瓶颈制约。土地紧张、融资不畅是制约我县经济社会发展的两大突出矛盾，必须引起高度重视。国家实行最严格的土地政策绝非权宜之计，不能心存幻想。要正确处理“保护耕地”与“保障用地”的关系，做到集中指标保重点，一般项目靠挖潜。抓好项目包装，积极跑办，争取更多的项目进入国家和省重点计划笼子，以项目争取土地指标；对近几年的开工项目来一次全面清理，对长期只占不用的，依法收回用地指标；对关停企业、废旧砖窑等，进行疏理整合，挖掘土地潜力。加强银企对接，为项目建设提供资金支持。二是用劲招商引资。招商引资是项目建设的生命线。要进一步强化招商引资目标责任制，建立、健全以政府为主导、企业为主体、全民共同参与的招商引资机制，形成招商整体合力。突出开展好京津、苏浙等地的阵地招商。下大力抓好软环境建设，全力为企业发展创造良好的外部环境。树立“三分招商，七分安商”的理念，促进招引企业发展壮大，实现一次招商、二次重组、多次嫁接、裂变发展。今年力争引进投资500万元以上的项目20个，实际利用外资560万美元。三是强化督促服务。加强重点项目跟进服务，实施重大项目集中调度、责任包干、联审联动、严格奖惩、督查督办五项推进制度，促进在建项目按时履约，按约投资，尽快投产见效。

第三、强力推进新农村建设，夯实跨越发展的基础

始终把解决好“三农”问题作为工作的重中之重，以工业化富裕农民，以城镇化带动农村，以产业化提升农业。加快建立健全以工促农、以城带乡的政策体系，形成城乡良性互动的发展格局。

发展新农业。积极推广优质小麦、玉米、棉花等农业适用技术，良种覆盖率提高到95%以上，启动农业有害生物预警与控制区域站建设，加快实施测土配方施肥项目，粮食总产量稳定在32万吨。加快发展果品业，坚持区域化布局、标准化生产、产业化经营、品牌化销售，积极培育优质果品基地；以方田林网和通道绿化为重点，抓好植树造林，深化林权改革，全年植树1万亩100万株。加强奶牛、特种养殖优良品种的引进和疫病防控，促进畜牧业健康发展，力争年产鲜奶3500吨，特种养殖年出栏46万只。加大对龙头企业的扶持力度，重点引导益海粮油、正阳红花生油、茂源养殖小区、天翼特养场等一批有潜力、有规模的企业做大做强，有效发挥龙头带动作用。大力发展各类农民专业合作组织，争取年内新增6个以上。加强农业生产标准化和质量安全体系建设，继续鼓励申报农产品无公害、绿色食品认证，对申报成功的单位给予奖励。

建设新家园。以搞好规划、加强基础设施建设、整治环境为重点，深入开展文明生态村创建工作。在巩固提高一、二批116个村创建活动的同时，今年再完成30个村的创建任务，逐步搭建起“十”字架型文明生态长廊。在做好皮草环线12.4公里油面铺设和全部路基处理工程的同时，力争完成102个村87.8公里通村水泥路建设，全部实现村村通，建立健全乡村公路管护机制，做好大（庆）广（州）高速公路建设配合工作，积极跑办魏广路、肖马路，力争早立项、早建设，逐步构建起主干相通、支线相连、纵横交织、便捷顺畅的大交通网络。进一步完善农田水利基础设施，加快防洪排涝和引蓄水配套工程建设。新建改建桥涵23座，节制闸2座，打更新井350眼，新铺防渗管道10万米，新增节水防渗面积4万亩。

构建新机制。认真落实粮食直补、良种补贴、农机补贴等各项惠农政策。积极推进补贴资金支付方式改革，实行“一折通”，确保及时、足额发放到位。坚持公共财政向“三农”倾斜、公共设施向农村延伸、公共服务向农民覆盖，促进城乡协调发展。稳定并完善农村土地承包关系，依法推进土地使用权流转，发展土地适度规模经营。认真落实“一事一议”政策，引导农民积极开展基础设施建设，加快发展公益事业。

培育新农民。坚持把培育新型农民作为新农村建设的基础性工程，通过提高农民的文化素质和致富能力，为增产增收和改变乡容村貌提供有力的人才保障。抓好务工农民队伍建设，强化劳动技能和技术培训，扩大劳务输出，力争全年向外输出人员1万人次。抓好务农农民素质提高，加大职业教育力度，培育有文化、懂技术、会经营的现代新型农民。积极推进农村精神文明建设，大力开展“讲文明讲卫生讲科学树新风”活动，倡导健康、文明、科学的生活方式，促进农村和谐发展。

第四、奋力搞好城市建设，改善跨越发展的条件

城市是一张名片，代表着一个地方的发展水平和文明程度。把县城规划建设管理好，是时代的要求、人民的企盼。我们的目标是：奋战三五年，把县城建设成为独具特色和魅力的国家级环境优美城市。总体思路和要求是：打造“三个一”，精心组织实施好“五项工程”。“三个一”，即“一带、一区、一园”。“一带”就是打造以卫千渠为轴心，贯穿县城的一条靓丽景观带；“一区”就是打造以城市花园为中心，配套完善、功能齐全的现代化新城区；“一园”就是打造以千亩森林公园为核心，适宜人们游玩、休闲、娱乐的观光游乐园。“五项工程”：一是卫千渠蓄水工程。加快卫千渠人民桥、新华桥、平原桥、胜利桥等四座桥的拓宽改造，启动完成人工湖改造和城北节制闸建设，为今冬蓄水创造条件。加快卫千渠县城段两侧硬化、绿化、亮化工程，逐步打造局部都市景观。二是城区路网工程。完成平原街、胜利北路、人民街东关段拓宽及便道硬化、绿化、亮化工程，完成市场西街、朝阳西街等7条小街小巷建设，启动西、北、南三个出口的绿化包装。三是新区重点建设工程。启动第四中（小）学和枣强县文体活动中心建设，力争完成当代城市花园二期工程，进一步提升县城品位。四是森林公园三期工程。完成漫步道铺设、广场铺装和董子亭建设，新植、补植花木20多个品种6万株。五是市政基础设施工程。加快集贸市场、供水管网、垃圾池、垃圾箱、公厕规划建设，进一步完善县城功能，提升县城品味。

城市发展，三分建，七分管。必须进一步加强县城规划与管理。采取多种有效形式，加强对县城总规和控制性详规的宣传教育，进一步增强市民的规划意识、参与意识和配合意识，使规划深入人心，在全社会形成自觉遵守规划、自觉执行规划的良好氛围。坚决维护规划的科学性和权威性，对不符合县城规划的建设项目，一律不予审批；对破坏县城规划的建筑，坚决予以拆除，切实做到“规划一张图、审批一支笔、建设一盘棋”。积极探索城市建设多元投入机制，鼓励社会各界参与县城建设。全面提高城市管理水平，成立城市管理综合执法办公室，进一步明确职责，建立严格的监督、监察机制，加强动态管理和精细化管理，开展好建筑市场、广告牌匾、环境卫生、道路交通等专项集中整治行动。研究建立“管理手段法制化、管理机制市场化、管理过程社会化”的长效机制，努力营造秩序井然、整洁优美的城市环境。

大营等建制镇继续按照“依托产业、形成特色、重点突破、有序推进”的原则，搞好基础设施建设，完善功能，提升品味，打造亮点，聚集要素，加快向现代化小城镇迈进，进一步强化对周边地区的辐射带动效应。

城市发展要以服务业为支撑。服务业的发展水平是衡量现代社会经济发达程度的重要标志。要把服务业发展摆上政府工作的重要议程，要以中国大营国际皮草交易中心为龙头，加快发展生产性服务业；以益海粮油为中心，积极发展物流、信息、咨询等现代服务业；以规模住宅小区为重点，规范发展房地产业。全力打造商贸繁荣、流通兴旺、人气鼎盛、活力迸发的新枣强。

第五、努力做好财税工作，稳固跨越发展的保障

受税案影响，今年我县的财税工作面临前所未有的压力，必须将财税工作作为政府的首要任务，一手抓增收，一手抓节支，努力将影响降到最低程度。

要进一步加大税源培植力度。加快发展是增加财政收入的根本出路，而要增加财政收入，难点在皮毛，重点也在皮毛；潜力在皮毛，出路也在皮毛。因此，必须把皮毛业的发展作为重中之重，一方面要全力抓好对现有一般纳税人的规范治理；另一方面要积极培育新的经济增长点，加强对小规模纳税人、零散业户的管理，促其发展壮大，成为新的一般纳税人。同时，积极为玻璃钢、机械制造业创造良好的税收环境，最大限度地防止税源流失。

要进一步加大税收征管力度。进一步完善以征收、管理相制约，以申报、稽查相结合的征管体制。加强重要税种的有效监控，切实做到依法征管，据实征收，确保应收尽收，均衡入库。建立健全非税收入征管新机制，增强政府宏观调控能力。进一步完善协税护税网络，千方百计为税收执法创造良好的外部环境。进一步加强法律政策宣传，加强企业负责人和会计培训，教育引导广大经营业户牢固树立诚信意识、法制观念，规范自身行为，依法照章纳税。

要进一步加大支出优化力度。严格控制一般性开支，今年的公用支出比去年降低20%，以确保工资正常发放，确保机关正常运转，确保社会稳定。建立健全财政专项资金审计制度，强化政府采购管理，加强对资金使用情况的监督检查，严肃查处违规违纪行为，不断提高理财水平。

要进一步加大考核奖惩力度。按照上级要求，调整完善乡镇财政管理体制，强化对国税、地税、财政部门的考核奖惩。充分调动乡镇和部门发展经济、培植财源的积极性，确保全面完成今年的财政收入任务。

第六、大力加强和谐枣强建设，提升跨越发展的质量

构建和谐枣强，是实现富民强县、全面建设小康社会的内在要求，也是全县人民的共同愿望。必须坚持以人为本，关注民生、维护民利，尽最大努力实现好、维护好、发展好最广大人民群众的根本利益，让全县人民更多地享受改革发展成果，促进人的全面发展和社会更加文明和谐。

做好就业再就业工作，切实筑牢民生之本。广辟就业渠道，完善和落实扶持自主创业的政策措施，鼓励和推动全民创业。高度重视下岗职工、零就业家庭、“4050”困难群体、高校毕业生和退伍军人的就业工作。全县新增就业岗位1200个，帮助200名下岗失业人员实现再就业，城镇登记失业率控制在3%。

抓好社会保障体系建设，切实夯实民生之基。完善城镇职工基本养老、医疗和失业、工伤、生育保险制度。抓好民营企业职工参保试点工作，积极扩大社会保险覆盖面。加强社保资金监管，确保资金专款专用、安全运行。加强城镇居民、农村村民最低生活保障和“五保”供养管理，逐步建立失地农民社会保障制度，解除群众后顾之忧。启动建设区域性敬老院，关心支持老龄事业、残疾人事业。努力构建常态救助、紧急救援和社会互助三大救助系统。

加快社会事业发展，切实构建民生之路。积极推进科技进步，努力创建机制、体系、服务三大科技创新平台。进一步加强与河北农大、河北科大、陕西科大等院校的县校合作，加快新技术、新工艺、新成果的引进、推广、转化，全年力争引进新技术、新成果8 项，科技贡献率达到52 %以上。优先发展教育事业，实施“两免一补”工程，全部免除义务教育阶段学生学杂费，取消小学、初中服务性收费和代收费。稳步推进学校布局结构调整，启动建设第四中（小）学。大力实施素质教育，加快推进普及高中阶段教育。以市场为导向，整合职业教育，更多地培养实用人才。深化新型农村合作医疗，努力提高参合率。加强医院、卫生院、社区服务站三级网络建设，改善医疗条件，提高医疗水平。健全突发公共卫生事件应急机制，不断提高疾病预防和控制能力。稳步推进有线电视村村通试点工程，努力提高有线电视覆盖率。

维护社会稳定，切实铸坚民生之盾。坚持依法治访，严格落实信访工作领导责任制和责任追究制。畅通群众诉求渠道，确保群众话有处说、怨有处诉、难有处解、事有处办。加强社会治安综合治理，继续开展“打黑除恶”专项斗争，严厉打击各类违法犯罪活动。扎实推进“五五普法”和依法治县工作，积极构建人民调解、行政调解、司法调解“三位一体”矛盾纠纷调解工作体系，最大限度化解各类矛盾。高度重视安全生产工作，坚持“安全第一、预防为主”，加强监管、深入排查、消除隐患，坚决遏制重特大事故发生。强化食品、药品监管，保障人民群众健康安全。

促进人与自然和谐发展，切实塑造民生之美。随着我县工业化进程的加快，环境约束的矛盾越来越突出。环境污染，不仅危害人民群众健康，引起群众不满，而且也难以实现可持续发展，已经到了非下决心解决的时候了。要按照“在发展中保护、在保护中发展”的方针，限期治理一批污染企业，对经过治理仍然不达标的坚决依法关停取缔。要大力发展循环经济，加快大营污水处理厂的建设进度，着手启动县城污水处理厂建设，努力建设资源节约型、环境友好型社会。继续开展文化、科技、卫生三下乡、三扎根，深入开展向林秀贞学习活动，大力推进和谐家庭、和谐企业、和谐单位、和谐社区建设。积极开展城乡爱国卫生活动，创造整洁优美的生产生活环境。切实做好人口与计划生育工作，人口自然增长率控制在6.5‰以内。

此外，认真做好国防动员、统计、物价、档案、邮政等工作。

四、切实加强政府自身建设

站在新的历史起点，我们肩负的责任十分重大。必须按照“为民、务实、清廉”的要求，坚持按县情谋事，按民意定事，按政策办事，按规律干事，按规矩处事，努力建设负责任、有作为、人民满意的政府。

一要自觉提高与时俱进求创新的能力，加快建设学习型政府。面对日新月异的形势变化，面对全面建设小康社会的艰巨任务，我们必须正视自身存在的知识危机、本领危机和岗位危机，深入学习邓小平理论和“三个代表”重要思想，切实加强政治、经济、文化、科技、法律等知识的学习，不断提高科学决策、领导发展的能力，廉洁勤政、依法执政的能力，掌握技能、服务群众的能力，调处矛盾、维护稳定的能力。要把加强理论学习同实践锻炼有机地结合起来，同改进工作方法、转变思维方式紧密地结合起来，学会在实践中博采众长、提高素质，学会运用科学的方法和辩证的思维谋划发展、推动工作。要围绕跨越发展这个主题，深入调查研究，不断研究新情况，探索新思路，解决新问题。

二要全面提高求真务实强服务的能力，加快建设服务型政府。以“解放思想、加快发展”大讨论活动为契机，大力倡导胡总书记提出的“八个方面良好风气”，做到为民、开拓、务实、清廉，增强法律纪律约束力、政策决策执行力和政府公信力。引导广大干部用心想事，用心谋事，用心干事，在“坚持科学发展、构建和谐枣强”的实践中培育良好的作风。进一步转变政府职能，提高经济调节和市场监管水平，强化公共服务和社会管理职能。鼓励创新，鼓励改革，鼓励进取，旗帜鲜明地为创新者壮胆，为改革者护航，为进取者助威，努力营造“创新实干、跨越发展”的浓厚氛围。强化机关效能建设，增强政府公务员的服务意识，降低行政成本，提高行政效率。

三要切实提高勤政为民抓落实的能力，加快建设责任型政府。坚持以人民为主人、视人民为亲人、尊人民为老师，在政治上代表群众，感情上贴近群众，行动上服务群众，赢得群众的信任和支持。坚持不懈地为民办实事，完善为民办实事的长效机制，突出抓好关系群众切身利益的10件实事。进一步加强廉政建设，严厉查处各类违法违纪案件，严格整治商业贿赂，坚持从完善体制、制度入手，进一步规范事权、财权和人事权，实施政府“阳光工程”，切实提高政府采购、工程招投标等行为的透明度，努力从源头上遏制腐败。强化审计监督和行政监察，建立健全政府投资责任制和责任追究制，全面开展绩效评估。加强政风建设，严格规范和控制各类检查、评比、达标活动，进一步改进会风文风。

四要重视提高依法行政促规范的能力，加快建设法治型政府。深入实施《行政许可法》、《公务员法》、《公务员处分条例》，依法规范行政权力，不断提高依法行政水平。强化对政府部门、行政执法机构行使权力、履行职责、执行程序的监督检查，切实规范行政执法行为，增强政府执行力和公信力。进一步完善重大事项社会公示制度、社会听证制度和专家咨询制度，推进政府决策的科学性、民主性。认真执行人大及其常委会的各项决议、决定，主动支持政协履行政治协商、民主监督、参政议政的职能。自觉接受社会监督和舆论监督。加快推进“法治枣强”建设，全力打造民主、公正的法治环境。

各位代表！枣强正处在实现跨越的关键时期，新的目标令人鼓舞，新的形势催人奋进，新的征程任重道远。让我们在中共枣强县委的坚强领导下，高举邓小平理论和“三个代表”重要思想伟大旗帜，以更加饱满的热情、更加昂扬的斗志、更加创新的精神、更加务实的作风，同心同德、锐意进取、扎实工作，为枣强的早日崛起而努力奋斗！
